# 526 Methamphetamine Intoxication Predicts Worse Outcomes in Severe Frostbite Injury

**DOI:** 10.1093/jbcr/irae036.161

**Published:** 2024-04-17

**Authors:** Michelle M Rayne, Nicholas J Larson, Abigail Rhone, Alexandra M Lacey

**Affiliations:** University of Minnesota Medical Center, Savage, MN; Regions Hospital, St Paul, MN; Regions Hospital, Minneapolis, MN; Regions Burn Unit, Saint Paul, MN; University of Minnesota Medical Center, Savage, MN; Regions Hospital, St Paul, MN; Regions Hospital, Minneapolis, MN; Regions Burn Unit, Saint Paul, MN; University of Minnesota Medical Center, Savage, MN; Regions Hospital, St Paul, MN; Regions Hospital, Minneapolis, MN; Regions Burn Unit, Saint Paul, MN; University of Minnesota Medical Center, Savage, MN; Regions Hospital, St Paul, MN; Regions Hospital, Minneapolis, MN; Regions Burn Unit, Saint Paul, MN

## Abstract

**Introduction:**

The best treatment for severe frostbite is thrombolysis with or without vasodilators, which allows reperfusion of the damaged tissue. Methamphetamine is a potent vasoconstrictor. While there is data in the burn population that methamphetamine use is associated with worse outcomes, there is no current literature on this topic in frostbite patients. It was therefore predicted that methamphetamine use predicts worse outcomes in severe frostbite injury.

**Methods:**

This was a retrospective, single-center, observational study at a Midwest burn center. Inclusion criteria were ≥14 years of age at time of admission, severe frostbite (defined as frostbite treated with intra-arterial (IA) thrombolytics), and >24hr of IA thrombolytics as an infusion. Patients were excluded if they were lost to follow up. Over a 15 year period, 165 patients met these criteria.

The primary outcome evaluated was salvage rate, using the Hennepin Frostbite Score. To obtain this score, surgeons assessed the initial angiography images to assign a score for the amount of tissue at risk (R). A surgeon then evaluated the patient’s operative notes to assign a score for the amount of tissue amputated (A). Salvage score (S) was then calculated as follows: S = R-A. Salvage rate was calculated using this score as follows: Salvage rate = (S / R)*100%.

**Results:**

In this sample of patients (N=165), 23.6% were positive for methamphetamine on admission (N=39). Using chi-square analysis, patients who were methamphetamine positive were statistically significantly more likely to have an amputation than those who were not (P=0.0132). All patients who had a 0% salvage rate were methamphetamine positive. This was not statistically significant.

**Conclusions:**

We found that all patients with a 0% salvage rate were methamphetamine positive. This was not statistically significant given the low N of 11, however we find this data clinically significant and represents potential evidence of a statistical association.

This study was limited by low sample size, and thus was lacking in power to predict outcomes on a large scale. Given that this is the only study to risk stratify the relationship between methamphetamine use and salvage rate, we believe this should prompt further investigation into this relationship to evaluate for statistical significance on a larger sample size.

**Applicability of Research to Practice:**

If this relationship is again identified in a study with a larger sample size, this would indicate a need for investigation into the most effective treatment for this population and potentially indicate a need for more aggressive intervention.

